# Evidence from neuroimaging for the role of the menstrual cycle in the interplay of emotion and cognition

**DOI:** 10.3389/fnhum.2013.00374

**Published:** 2013-07-24

**Authors:** Julia Sacher, Hadas Okon-Singer, Arno Villringer

**Affiliations:** ^1^Clinic for Cognitive Neurology, University LeipzigLeipzig, Germany; ^2^Max Planck Institute for Human Cognitive and Brain Sciences, Cognitive NeurologyLeipzig, Germany; ^3^Department of Psychology, University of HaifaHaifa, Israel

**Keywords:** menstrual cycle, neuroimaging (anatomic and functional), emotion-cognition interaction, mood, emotion regulation, sex hormones, reward

## Abstract

Women show increased predisposition for certain psychiatric disorders, such as depression, that are associated with disturbances in the integration of emotion and cognition. While this suggests that sex hormones need to be considered as modulating factors in the regulation of emotion, we still lack a sound understanding of how the menstrual cycle impacts emotional states and cognitive function. Though signals for the influence of the menstrual cycle on the integration of emotion and cognition have appeared as secondary findings in numerous behavioral and neuroimaging studies, this has only very rarely been the primary research goal. This review summarizes evidence: (1) that the menstrual cycle modulates the integration of emotional and cognitive processing on a behavioral level, and (2) that this change in behavior can be associated with functional, molecular and structural changes in the brain during a specific menstrual cycle phase. The growing evidence for menstrual cycle-specific differences suggests a modulating role for sex hormones on the neural networks supporting the integration of emotional and cognitive information. It will further be discussed what methodological aspects need to be considered to capture the role of the menstrual cycle in the emotion-cognition interplay more systematically.

## Introduction

Sex hormones have been shown to influence emotional states and cognition (Schmidt et al., 1998; Bloch et al., 2000). This is supported by a wide body of animal data and reflected in diverging prevalence rates for men and women for many psychiatric diseases that are associated with cognitive biases to emotional information, such as depression and anxiety (Soares and Zitek, 2008). While hormonal transitions across the life-span represent periods of heightened vulnerability for development of mood disorders for women, the prevalence rates for depression between the sexes are most prominent during the reproductive years. The most extreme changes in gonadal hormones, such as the postpartum period, have consistently been reported as a time of increased risk for depression (O'Hara, 2009). The menstrual cycle offers a unique opportunity to study whether subtle fluctuations of sex hormones can influence neuronal circuits implicated in the cognitive regulation of emotional processing.

The menstrual cycle can be divided in a follicular and a luteal phase (Terner and De Wit, 2006): the follicular phase is used generally to refer to the period after completion of menses until ovulation. During menses and early in the follicular phase, levels of both progesterone and estrogen are very low, while toward the middle and later portions of the follicular phase estrogen levels begin to rise (Griffin and Ojeda, 2004). During the luteal phase, the period between ovulation and menses-onset, estrogen levels decrease to a moderate level until they fall sharply just before the onset of menstruation. Progesterone levels rise after ovulation, peak at the mid-luteal phase, and fall rapidly just before menstruation (Griffin and Ojeda, 2004; Terner and De Wit, 2006). Most studies addressing menstrual cycle dependent changes compare an assessment during the late follicular phase (when estrogen levels are high and progesterone levels low) and during the late luteal phase (when estrogen levels are low and progesterone levels high).

It has been established that sex hormones act on the central nervous system and influence the organization of neural circuits during the prenatal period (Collaer and Hines, 1995). Sex hormones are known to directly influence the hypothalamus and the hippocampus: areas that are implicated in emotional processing, perception and memory, as well as in the interpretation of sensory information (Fanselow and Dong, 2010; Hines, 2010). As it becomes clearer that hormonal transition periods across the life span also affect brain organization, some neuroimaging studies have started addressing the relevance of subtle hormonal fluctuations across the menstrual cycle on brain architecture and connectivity. However, in most cases, the menstrual cycle is entered to statistical analyses as a nuisance regressor (Lonsdorf et al., 2011), or controlled for by only testing male samples (Karama et al., 2011).

In the few instances that the menstrual cycle phase was the primary research aim, typically the research focused on cognitive domains, using mental rotation or language tasks (Masters and Sanders, 1993; Fernandez et al., 2003; Schoning et al., 2007; Pletzer et al., 2011). Few studies investigated the impact of the menstrual cycle on the interplay of emotion and cognition. This lack of knowledge is striking, considering the many interwoven aspects of emotion and cognition. The findings summarized hereafter (for a detailed overview see Table 1) show that menstrual cycle phase affects the reaction to emotional stimuli and reward, as evidenced by behavioral biases in reaction time and neural activation. In line with this evidence, the menstrual cycle also appears to impact a neural network implicated in cognitive control of emotion. This evidence suggests that the menstrual cycle should be considered as a modulating factor when examining the behavioral and neural response to emotional information. The unique combination of sex hormones in different phases of the menstrual cycle may assist in furthering our understanding of inter- and intra-individual differences in emotional reactions.

**Table 1:** **Summary of imaging studies exploring the impact of the menstrual cycle on neuroplastic changes of relevance to the interplay of emotion and cognition**.

**Study**	**Number of subjects**	**Design**	**Time of menstrual cycle assessed**	**Imaging modality**	**Main findings**
Protopopescu et al., 2008a,b	21	Within-subject, two time-points per subject	Late follicular phase	VBM-MRI	Right anterior hippocampus (GM-increase)
			Late luteal phase		Right dorsal basal ganglia (GM-decrease)
Tu et al., 2010	32 vs. 32	Healthy control group vs. PDM subjects	Peri-ovulatory phase	VBM-MRI	Medial prefrontal cortex (mPFC), insula (GM-decrease).
					Anterior/dorsal posterior cingulate cortex (ACC/dPCC), hippocampus, hypothalamus, (GM-increase)
Hagemann et al., 2011	8	Within-subject, two time-points per subject	Early follicular	VBM-MRI	Global GM-volume increase, volume loss in CSF during ovulation
	Note: association with estradiol found in 7 women		Mid-luteal phase		
Dreher et al., 2007	13 healthy regularly cycling women	Within-subject, two time-points per subject	Mid-follicular	fMRI during a monetary reward task	Enhanced activation in the amygdala and the OFC during mid-follicular;
			Mid-luteal		Enhanced activation in the DLPFC and the dACC during mid-luteal
Protopopescu et al., 2008a,b	8 PMDD; 12 asymptomatic women	Within-subject, two time-points per subject	Late-follicular	fMRI during a Go/No-go task	Late luteal vs. late follicular: PMDD women showed reduced activation in medial OFC and ventral striatum, and enhanced activation in the amygdala and the lateral OFC, compared to healthy controls
			Late-luteal		
Jacobs and D'Esposito, 2011	24 healthy regularly cycling women	Within-subject, two time-points per subject	Early follicular	Behavioral and fMRI	COMT activity has been shown to drive the direction of the effect estrogen had on working memory
			Late-follicular		
Ossewaarde et al., 2011	28 healthy regularly cycling women	Within-subject, two time-points per subject	Late-follicular	fMRI during a delayed incentive monetary reward task	Enhanced ventral striatal activation in the late luteal compared to the late follicular phase
			Late-luteal		
Mareckova et al., 2012	10 healthy regularly cycling women	Within-subject, two time-points per subject	Early follicular (perimenstrual)	fMRI during passive viewing of faces (angry vs. moving circles; ambiguous faces vs. moving circles)	Stronger BOLD response to angry faces in the right FFA, left IFG, left temporal gyrus; and to ambiguous faces in the right STS, bilateral IFG, right lingual gyrus, in late follicular compared to early follicular (perimenstrual) phase
			Late-follicular		
Reiman et al., 1996	10	Within-subject, two time-points per subject	Mid-follicular phase	FDG-PET	Higher glucose metabolism in thalamus, prefrontal, temporo-parietal, inferior temporal cortex
			Mid-luteal phase		Higher glucose metabolism in superior temporal, anterior temporal, occipital cortex, cerebellum, cingulate, anterior insula

Note: CSF, cerebral spinal fluid; dACC, dorsal anterior cingulate cortex; DLPFC, dorsolateral prefrontal cortex; FDG, [^18^F]-fluorodeoxyglucose; fMRI, functional magnetic resonance imaging; GM, gray matter; OFC, orbitofrontal cortex; FFA, fusiform face area; IFG, inferior frontal gyrus; STS, superior temporal sulcus; PDM, primary dysmenorrheal; PET, positron emission tomography; PMDD, pre-menstrual depressive disorder; VBM, voxel based morphometry.

## Neuroplastic changes in the human brain across the menstrual cycle

As evidence for short-term modification of brain plasticity is growing, we continue to adapt our understanding of how brain structure is organized throughout the lifespan. With proliferating documentation supporting a substantially less rigid architecture of the brain than previously hypothesized, identifying the mechanisms that drive neuroplastic modification has become a major focus of interest. Among those factors that are discussed to induce such neuroplastic changes are deliberate training (Draganski et al., 2004), exercise (Taubert et al., 2011), stress (Liston et al., 2009), as well as hormones (Baroncini et al., 2010).

### Menstrual cycle dependent changes in structural and functional connectivity: impact on emotion and cognition interaction

The subtle hormonal fluctuations induced by the menstrual cycle have been explored as potential neuroplastic factors in a few neuroimaging studies at rest. A voxel based morphometry (VBM) study comparing women suffering from cyclic menstrual pain with peri-ovulatory cycle-matched healthy women found substantial brain morphological changes in brain regions implicated in pain transmission but also in affect regulation and top-down modulation of negative affect including the medial prefrontal cortex (mPFC), the anterior/dorsal posterior cingulate cortex (ACC/dPCC), hippocampus, hypothalamus and insula (Tu et al., 2010). A pilot within-subject positron emission tomography (PET) study reported significantly higher glucose metabolism for the mid-follicular menstrual cycle phase in thalamic, prefrontal, temporo-parietal and inferior temporal regions whereas during the mid-luteal menstrual cycle phase increased glucose metabolism in superior temporal, anterior temporal, occipital, cerebellar, cingulate and anterior insular regions was found (Reiman et al., 1996). In a second preliminary VBM study, a change in overall brain size according to menstrual cycle phase, more specifically an increase in gray matter and loss of cerebral spinal fluid (CSF) during the time of ovulation, was found (Hagemann et al., 2011). This brain volume change could be associated with progesterone levels and also correlated, after excluding one outlier, with the estradiol rise prior to ovulation. A volumetric MRI study including twenty-one women in an intra-individual design reported an increase in the right anterior hippocampus in the late follicular versus the late luteal menstrual phase (Protopopescu et al., 2008a). The hippocampus has been implicated in self-referencing during recall and prospection (Muscatell et al., 2010), the formation of emotional memories (Eisenberger et al., 2007) and the processing of facial expressions (Critchley et al., 2000; Fusar-Poli et al., 2009). Traditionally often referred to as the “memory-region,” the hippocampus has recently been discussed as a crucial integrator of emotion and cognition (Small et al., 2011). Particularly the caudal/ventral hippocampal region (corresponding to anterior in primates) has been linked to controlling the hormonal stress response via the hypothalamic-pituitary-adrenal axis. In addition, smaller hippocampal size and deficient function were related to psychopathologies characterized by maladaptive emotional behavior, such as depression, post-traumatic stress disorder and bipolar disorder, whose drug treatment impact hippocampal structure and function (Fanselow and Dong, 2010).

Proptopopescu and colleagues (Protopopescu et al., 2008a) further reported a volumetric decrease in the dorsal basal ganglia during the late follicular menstrual phase. A trend for a neurochemical change in the basal ganglia was observed to fluctuate with the menstrual cycle as a secondary finding in a PET-study exploring sex- and age-differences in dopamine receptors: lower D2 binding in the late follicular menstrual cycle phase was detected but did not meet the threshold for statistical significance (Wong et al., 1988). While the authors acknowledged the preliminary character of their dataset comprised of six healthy women, they made the interesting point that the signal observed was present in each of the six subjects.

### Changes in the reward-related neural system across the menstrual cycle

Dopamine represents a key regulator in the integration of cognitive and emotional information processing in the basal ganglia and has been implicated in synaptic plasticity. If these preliminary findings can be replicated in a larger sample, this would argue for the menstrual cycle to impact a major neurochemical axis relevant to numerous neuropsychiatric diseases that display sex-disparity, such as attention deficit disorder, schizophrenia, addiction, and Parkinson's. Recent fMRI results corroborate the link between dopamine and the menstrual cycle: performance in a working memory task increased with dopaminergic transmission rate (indicated by catechol-O-methyltransferase, COMT, enzyme activity) in the late follicular phase but decreased with dopaminergic transmission rate in the early follicular phase and could be predicted by activation of PFC in both conditions (Jacobs and D'Esposito, 2011). These findings suggest that the hormonal fluctuations caused by the menstrual cycle set the stage for a dynamic modulation of cognition and emotion by dopaminergic transmission.

In addition to the above-mentioned roles in cognitive and emotional processes, dopamine is involved in mediating reward. To directly examine the notion that the reward system is influenced by menstrual cycle phase, a study exploring monetary reward in a counter-balanced intra-individual design collected fMRI data in thirteen healthy women (Dreher et al., 2007): The authors found greater blood-oxygen-level-dependent (BOLD) response in the amygdala, the orbitofrontal cortex (OFC), midbrain and the striatum during the mid-follicular phase, brain areas that are highly inter-connected both anatomically and functionally and that are key for autonomic control, emotional processing and reward. A possible interpretation of these findings may be a more responsive reward system shortly before ovulation. Complementary neuroimaging work on the dopaminergic system in 28 healthy women also revealed differences in mesolimbic incentive processing at distinct times of the menstrual cycle (Ossewaarde et al., 2011). Applying a monetary reward incentive delay task, the authors could show an enhanced ventral striatal response in the late luteal versus the late follicular phase and suggest that changes in functioning of mesolimbic incentive processing circuits may underlie premenstrual increases in normal and abnormal motivated behaviors such as food and drug cravings (Ossewaarde et al., 2011).

### Cycle-dependent biases in emotional control

As noted, Dreher et al. (2007) found altered functional activity in the DLPFC and dACC during the mid-luteal phase. These brain regions have important roles in the control of emotional interference to cognitive performance. Recent findings (Mareckova et al., 2012) report stronger BOLD fMRI responses during passive viewing of ambiguous and angry faces (compared to control stimuli) in neural regions related to emotion processing and control, when comparing the mid-cycle and the menstrual phases in freely-cycling women. These regions included the right superior temporal sulcus, bilateral inferior frontal gyrus (IFG), and the right lingual gyrus for ambiguous faces, and the right fusiform face area (FFA), the left IFG, and the left middle temporal sulcus for angry faces. Stronger activation in the right FFA was also found when comparing women taking oral contraceptives compared to controls, and were further replicated in a group of 110 adolescent girls. In line with these findings, several studies showed an impact of the menstrual cycle on the ability to control emotional behavior. Specifically, it was suggested that biased processing of information during the late luteal phase facilitates symptoms of premenstrual depressive disorder (PMDD) (Cunningham et al., 2009). PMDD patients have been shown to demonstrate a luteal phase–dependent negative bias in facial emotion discrimination (Rubinow et al., 2007). This processing bias is in line with higher negative affect and impaired cognitive performance, particularly in memory tasks, in PMDD women during the late luteal phase (Reed et al., 2008). Protopopescu et al. (2008b) used an emotional modification of a Go/No-go inhibitory task in an fMRI experiment: women with PMDD showed enhanced processing of negative information, decreased processing of positive information, and diminished inhibitory control, especially in the context of negative information. Furthermore, these findings were accompanied by reduced activation in medial orbitofrontal cortex (mOFC) and ventral striatum, and enhanced activation in the amygdala and the lateral orbitofrontal cortex, in PMDD subjects versus controls, when comparing the late follicular and the late luteal cycle phases. These findings suggest reduced top-down inhibition of negative information in the late luteal phase in PMDD.

## Challenges and questions

Exacerbation of psychiatric illness has been associated with phases of steep sex hormonal fluctuations (Soares and Zitek, 2008). Several studies demonstrate the impact of such substantial hormonal change on several cognitive and affective domains (Greendale et al., 2010; Ladouceur, 2012; Workman et al., 2012). For the interaction between more subtle sex hormone fluctuations, such as the menstrual cycle, and mood-regulation, reports have been more controversial (Romans et al., 2012). However, one cannot draw the conclusion that the menstrual cycle has no role in the interplay of emotion and cognition from the observation that many studies in this emerging field have been underpowered or methodologically inconsistent. With regards to such methodological inconsistencies, it would be helpful to introduce to the field some sort of standardization to confirm regular menstrual cycles and ovulation of participants. Urine ovulation kits or blood samples demonstrating the pre-ovulatory LH/FSH surge could provide this information in order to ensure that subjects indeed go through the hormonal fluctuation characteristic of a regular menstrual cycle.

A major challenge reviewing the neuroimaging evidence for the impact of the menstrual cycle on brain regions is the lack of consistency in timing of assessment. With a few exceptions, most of the work reviewed did follow an intra-individual design comparing two or more menstrual cycle phases. Most studies included a comparison between follicular and luteal cycle phase, however both of these phases are approximately 12–14 days in length. As reviewed here, the choice to look at an early follicular time (when both estrogen and progesterone levels are low) or at a late follicular time (when progesterone levels are still minimal but estrogen levels are highest) is likely going to impact the results and can make it hard to evaluate data collected at different times.

One approach that has been taken by many in the endeavor to study the impact of sex hormones on the interplay of emotion and cognition is analyzing the correlations between hormone level and a neuroimaging parameter, such as changes in BOLD signal in emotional processing circuits. However, these reports tend to be difficult to interpret since a specific sex hormone can impact neurotransmitter-signaling differently in different states of hormonal environment. For example, data in rodents, primates, and humans have demonstrated that estrogen modulates behavioral sensitization to cocaine differently in the presence of progesterone than in the absence of progesterone (Evans and Foltin, 2010). The menstrual cycle thus provides a unique natural set-up to study the interactions of sex hormones in synergy and move beyond looking at simple correlations.

## Conclusion and perspectives

To summarize, studies have provided preliminary evidence for neuroplastic changes across the menstrual cycle, including the striatum, thalamus, hippocampus, insula, hypothalamus, amygdala, ACC, frontal cortex (OFC, DLPFC) and parietal areas. Most of these regions have substantial roles in the perception, processing or regulation of responses to emotional information. Menstrual cycle-dependent changes have also been demonstrated in reward-related behavior and to interact with dopaminergic transmission. Different patterns of neural activation have been found in women with clinical premenstrual mood change, also pointing to an influence of sex hormones on the neural activation related to cognition-emotion interaction. While the data are still sparse and substantial methodological differences have to be accounted for, it is likely that the subtle hormonal fluctuations that characterize the menstrual cycle modulate emotional behavior in women during their reproductive years.

The neural networks mediating cognition-emotion interactions are a topic of a long and on-going debate. Based on advanced analysis of neuroimaging data, Pessoa (2012) emphasized the interactions between evaluative and control sites as mediators of the impact of cognition on emotional perception. In line with this network view, effects of cognitive load on emotional processing were shown in fronto-parietal attention regions (Culham et al., 2001; Schwartz et al., 2005; Bishop, 2008; Tomasi et al., 2011) as well as limbic (Van Dillen et al., 2009) and sensory regions (Muggleton et al., 2008). Sex hormones are known to influence a number of neurotransmitters implicated in the regulation of cognition and affect, including acetylcholine, serotonin, dopamine, and norepinephrine (Genazzani et al., 1997; Mitsushima, 2010). Functional consequences of genetic polymorphisms in those neurotransmitter-systems need to be considered for the interaction between neurochemical environment and hormonal states in the brain. Genetic vulnerabilities for anxiety and depression in the serotonergic system (Lesch et al., 1996) may relate to the differential response across women to antidepressant treatment targeting the serotonin transporter. The enzyme metabolizing dopamine, catechol-O-methyltransferase (COMT), accounts for the majority of dopaminergic turnover in the PFC (Mannisto and Kaakkola, 1999). For COMT, carriers of the variant met/met (opposed to the met/val allele variant) showed better performance in an executive task and displayed enhanced PFC activation (Egan et al., 2001). Furthermore, COMT activity has been shown to drive the direction of the effect estrogen had on working memory (Jacobs and D'Esposito, 2011). The integration of epigenetic information to neuroimaging data across the menstrual cycle will be important in characterizing the functional consequences of genetic polymorphisms that are implicated in these neurochemical underpinnings of emotion and cognition.

Studies in healthy women during their reproductive years are no substitute for directly studying mood disorders associated with the menstrual cycle, such as PMDD. They do, however, provide an important framework to build upon as confounding factors like comorbidities can be excluded. For intervention studies it will be necessary to include clinical populations. The exploration of neural patterns in the emotional circuits that can be associated with techniques such as cognitive behavioral therapy (CBT) (Goldapple et al., 2004) and mindfulness stress reduction (Schwartz et al., 1996) in order to provide helpful alternatives or add-ons to psychopharmacological interventions in PMDD are promising new research directions. However, research on the menstrual cycle and the interplay of emotion and cognition has a broader scope than menstrual-cycle related disorders. An interaction of reproductive hormones and neuroplasticity has been reported for diseases that can generate abnormalities in emotional processing and social cognition, like multiple sclerosis (Tomassini et al., 2005) Alzheimer's (Pike et al., 2009) and migrane (Gupta et al., 2011). Furthermore, we know that treatment responses can differ immensely between the sexes, and within different hormonal states (Lukas et al., 1996; Justice and De Wit, 1999; Evans and Foltin, 2010).

In conclusion, the emerging research field of neuroimaging the menstrual cycle has already contributed many clinically relevant insights into powerful interactions between sex hormones and neural processes in emotion and cognition. While the majority of endocrinological neuroimaging research has focused on the role of estrogen on traditional aspects of cognition, more studies start to address the interwoven processing of emotion and cognition. The menstrual cycle provides a natural set-up to do so and it will be critical for the interpretation of studies across imaging centers to confirm the endocrine status on each test day of women undergoing a scanning protocol during their reproductive years.

### Conflict of interest statement

The authors declare that the research was conducted in the absence of any commercial or financial relationships that could be construed as a potential conflict of interest.
